# A critical analysis of the review on antimicrobial resistance report and the infectious disease financing facility

**DOI:** 10.1186/s12992-016-0147-y

**Published:** 2016-03-22

**Authors:** David M. Brogan, Elias Mossialos

**Affiliations:** LSE Health, Department of Social Policy, London School of Economics and Political Science, London, United Kingdom; Missouri Orthopaedic Institute, University of Missouri, Columbia, Missouri United States of America

**Keywords:** International Disease Financing Facility, Review on Antimicrobial Resistance, Antibiotic development

## Abstract

Over the past year, two major policy initiatives have been introduced focusing on stimulating antibiotic development for human consumption. The European Investment Bank has announced the development of the Infectious Disease Financing Facility (IDFF) and the British government commissioned the Review on Antimicrobial Resistance, led by Jim O’Neill. Each constitutes a major effort by the European community to address the evolving crisis of antimicrobial resistance. Though both have similar goals, the approaches are unique and worthy of consideration.

This manuscript utilizes a previously published framework for evaluation of antibiotic incentive plans to clearly identify the strengths and weaknesses of each proposal. The merits of each proposal are evaluated in how they satisfy four key objectives: 1) Improve the overall net present value (NPV) for new antibiotic projects; 2) Enable greater participation of Small to Medium Sized Enterprises (SME); 3) Encourage participation by large pharmaceutical companies; 4) Facilitate cooperation and synergy across the antibiotic market. The IDFF seeks to make forgivable loans to corporations with promising compounds, while the O’Neill group proposes a more comprehensive framework of early stage funding, along with the creation of a stable global market.

Ultimately, the proposals may prove complementary and if implemented together may form a more comprehensive plan to address an impending global crisis. Substantial progress will only be made on these efforts if action is taken at an international level, therefore we recommend consideration of these efforts at the upcoming G20 summit.

## Background

The prospect of a world without effective antibiotics is a daunting one. Some estimates suggest that deaths attributable to antimicrobial resistance may rise from the current estimate of 700,000 lives per year to ten million lives annually by 2050, at a cost to world GDP of US$100 trillion [1]. At the same time, almost 50 incentive strategies have been proposed to stimulate antibiotic development [2]. These include push, pull and hybrid mechanisms (combination of push and pull mechanisms), among many others, all with the ultimate goal of increasing the number of effective antibiotics for human consumption. The two most recent large scale efforts in this area are in the final stages of planning.

The first of these efforts, the Infectious Disease Finance Facility, is a funding mechanism proposed by the European Investment Bank (EIB) and the European Commission (EC) [3]. The second is an outline published by the Review on Antimicrobial Resistance, chaired by Jim O’Neill, which seeks to identify the characteristics of an optimal incentive strategy to promote private development of more antimicrobial compounds [4]. The O’Neill Review proposal is more comprehensive in its scope, but lacks a defined mechanism to implement its ideals. The EIB/EC financing facility outlines definitive steps, but is narrower in scope. Both potentially offer valuable contributions towards addressing the current crisis, however, each of these mechanisms utilizes slightly different strategies, and like any incentive plan, they are influenced largely by their country of origin, local politics, and specific agendas of the designers. Therefore, a framework is necessary to critically evaluate newly proposed strategies. We have recently described such a framework for evaluation of incentive mechanisms [5], and in this paper seek to apply this framework to the above initiatives.

The Infectious Disease Finance Facility (IDFF) was recently announced as a joint venture by the EC and EIB [3]. This facility will utilize risk sharing loans that will only require repayment if they result in a marketable product. If successful antibiotics pay money back into the fund, this will increase the overall size of the fund and allow further rounds of funding. The EC and EIB predict at least a 5x multiplier effect due to the requirement that grant recipients or other donors match at least half of the required funds for the project. While the exact terms of such loans have yet to be determined, each project applying for funding must have completed pre-clinical testing.

The Review on Antimicrobial Resistance chaired by Jim O’Neill, has written a preliminary report of their recommendations to spur antibiotic investment and development. Hereafter referred to as the O’Neill report, this documents asserts the need for development of two to four compounds with novel therapeutic profiles every decade in order to keep up with antibiotic resistance. The report specifically outlines three sets of necessary interventions:

1. Creation of a more predictable market for antibiotics to sustain commercial investment in R&D
2. Focused funding in early stage research (through a global AMR innovation research fund)
3. Innovations to promote efficient drug development

The O’Neill report notes the potential for early payment after development, or even during the development process, potentially as milestone payments or product development partnerships. While initially proposing a potential global buyer, the report recognizes the limited practicality of this scenario and instead advocates a hybrid model, including a lump sum payment as well as the option for companies to sell the developed drug for a profit. Early reports suggest that the group will advocate for US$2 billion to be allocated for push incentives, to fund projects in development through grants. Simultaneously, they would also allocate somewhere between US$16–37 billion for prize mechanisms [4]. The exact amount of each prize could vary depending on whether or not the purchaser will buy out the entire market for the pharmaceutical or just a portion of the market.

## Methods

While both of the above proposals have obvious merit, a closer analysis is warranted, and can be performed utilizing our previously proposed framework. This framework examines the requisite market criteria for a new incentive, as well as public health objectives and implementation feasibility.

### Market criteria

It is well recognized that the market fails for antibiotics, for a variety of reasons [6]. Therefore, an effective incentive strategy must correct these failures, in part by addressing four key criteria:

1. Improve the overall net present value (NPV) for new antibiotic projects
2. Enable greater participation of Small to Medium Sized Enterprises (SME)
3. Encourage participation by large pharmaceutical companies
4. Facilitate cooperation and synergy across the antibiotic market

#### Improve overall Net Present Value (NPV)

Potential compounds are evaluated based on their ultimate costs and revenues, as well as the likelihood of achieving returns. Returns are also discounted by the time cost of money, or the cost of the capital needed to fund the project. This complex calculation results in a net present value assigned to each project. Projects with high net present values – ideally demonstrating a high likelihood of returning significant profits – are likely to be pursued. Low or negative net present values (NPV) suggest that the costs are too high or the probability of achieving significant returns is too low.

Antibiotics generally suffer from low NPVs. One estimate puts the average NPV for antibiotics at US$-50 million, compared to US$1.15 billion for musculoskeletal drugs [7]. Therefore any mechanism that increases the NPV can incentivize antibiotic development. This can be done by improving the likelihood of approval, increasing profits or decreasing the costs. The likelihood of approval is a regulatory and safety issue, and strategies for this will be discussed later. Increasing profits could be accomplished by a lump sum payment mechanism, but any lump sum payment at the time of approval would be heavily discounted by the cost of capital over the time span needed to bring a drug to market, and by the overall low likelihood of achieving marketing approval. Put simply, uncertain money in the distant future would need to be promised in very large amounts to encourage concrete action and investment now. An alternative would be to lower costs, in part by subsidizing research. Mechanisms like this, termed push mechanisms, are not new, but they generally are not tied to an identifiable outcome and are sometimes criticized for lack of efficacy.

#### Enable greater participation of small to medium sized enterprises

Munos [8] found that the discovery of new molecular entities (NME) has remained relatively constant over the past 60 years, and suggests a correlation between the number of NME’s and the number of participating drug companies. He argues that a larger number of companies involved in research may speed up the acquisition of knowledge and lead to greater development. Small companies may not have the necessary funds to transition investigational compounds into clinical trials. Therefore, participation of these companies may be enhanced with a variety of mechanisms. Early stage funding could help bring their ideas to fruition, or at least allow them to collect more data to secure further funding from investors. Open source data sharing, utilization of public-private partnerships, patent buyouts and assistance with regulatory hurdles or global coordination of clinical trials are all mechanisms to help leverage the limited resources of smaller enterprises [2, 9]. An effective incentive will seek to include a mechanism to accomplish this.

#### Encourage participation by large pharmaceutical companies

The overall cost of development by a large pharmaceutical firm may be greater than $5 billion on average for each drug that makes it to market, and there is some evidence to suggest that this average is higher for larger pharmaceutical firms than smaller ones [10]. This is likely due to the fact that when smaller companies fail, they usually go bankrupt, while larger companies must carry the cost of the failure on their balance sheet. Still, it underscores the need for larger companies to allocate resources wisely and lessen their financial risks when pursuing projects with potentially limited payouts.

#### Facilitate cooperation and synergy across the antibiotic market

Success in antibiotic development largely aligns with public health goals, as discussed further below. Despite the financial risks involved, great potential exists for synergistic cooperation in development, regulation, distribution and monitoring. An ideal incentive mechanism would encourage cooperation among the various stakeholders, including local and national governments, international health organizations, industry leaders, as well as regulatory and medical personnel.

### Public health objectives (access and stewardship)

The optimal incentive will not only encourage participation and development of compounds via the four domains listed above, it will also seek to influence distribution and utilization of this unique resource. The efficacy of antibiotics is directly related to their utilization, so profligate prescribing may render them relatively impotent due to the emergence of resistant bacteria. High prices can restrict use, but also hinder appropriate access by those with less means. A delicate balance must be struck between judicious prescribing and ease of access regardless of income.

### Implementation feasibility

Multiple barriers will exist to implementation of any incentive scheme. Complex schemes may prove too wieldy or expensive, regulatory barriers may be too high, or trade agreements too rigid. Therefore the ease of implementation of any proposed incentive must also be considered in the final tally.

## Results

Having outlined a set of criteria by which to judge any proposed solution to stimulate antibiotic innovation, we may now critically compare the IDFF and O’Neill reports.

### Market criteria

The IDFF proposal would help to improve the NPV of a potential project by investing in the compound prior to market approval, likely in the clinical phase. Loan recipients must fund at least 25 % of the project costs, while the IDFF may fund up to half of the costs, with the remainder paid by third parties. Any funding during the development phase will improve the overall NPV, but funding in the later clinical stages will have a relatively smaller effect on the overall NPV calculation at the initiation of the project, given the time value of money. Therefore, the timing of investment in the IDFF proposal is a comparative weakness. So too is the overall amount of funding currently proposed. Initial estimates suggest that up to €300 million may be spread across a total of 9–12 projects. Development costs of new drugs have been projected to be as high as US$2.5 billion [11], although this number is somewhat debated and may vary depending on the exact accounting methods used [12]. Regardless, even if the true cost is half this, €300 million spread over ten projects may not be a sufficient investment. If the EIB assumes additional risk, the fund anticipates doubling the expected 5× multiplier to return up to a 10X multiplier from repayments, returns and co-investments, but it is unclear as to how this will be achieved.

In contrast, the O’Neill report advocates between US$18–35 billion in overall investment, a sum that dwarfs that proposed by the IDFF. Specifically, the investment could come in one of two phases: 1) early stage investment to support innovative, but riskier projects or 2) late stage lump sum payments at the time of, or within 1–3 years of, market approval. The lump sum payment would be designed to provide a sufficient market for the antibiotic to help ensure a de-linkage between the volume sold and the financial returns to the company. The exact mechanism by which this would be accomplished has not been determined, but could manifest as a global purchaser who would control the entire market for a designated antibiotic, or as a one off payment, with allowances for the company to continue to market the antibiotic. The advantage of this system is that the combination of early stage investment and late stage bulk purchasing can profoundly improve the NPV for a designated compound. The creation of an artificial market for the antibiotic could prove rather potent. Early stage investment will also provide more “bang for the buck”, and potentially decrease the sum needed at the time of market approval. Therefore, the O’Neill report recommendations will likely prove more effective in improving NPV for a potential project.

### Small to medium sized enterprise vs large pharmaceutical participation

The IDFF proposal anticipates allocating money to small and medium sized enterprises, approximately €25 million to each of 8–10 companies. These companies would be expected to fund up to 25 % of the project, with a maximum of 50 % of funds coming from IDFF money. The remaining funds could then be contributed by a third party. This could prove sufficient to allow initial testing of products. However, recent data suggests that pre-clinical testing costs on average may be as high as US$1.1 billion [11]. Therefore, the funding from IDFF would likely not be sufficient to fund development through pre-clinical testing – substantial additional support would be required. The IDFF report also suggests that the project should have completed pre-clinical testing, thus €25 million could only be applied to clinical testing.

The O’Neill proposal encourages SME participation through two separate mechanisms. The first is the creation of a global innovation fund to help support early stage research, this could specifically target neglected areas of research and encourage innovative thinking. The proposal suggests a sum of US$2 billion spread over 5 years. SME’s and large pharmaceutical firms alike could participate if their research was considered worthy of investment. The report suggests that the existence of the fund for 5 years would be sufficient to reinvigorate R&D spending for an additional 10–15 years. They also suggest that the fund should be paid for by contributions from the global pharmaceutical industry.

Secondly, the proposal advocates for more centralized platforms for clinical trials, as well as efforts to increase information sharing during early development to lessen the regulatory burden. This is a critical step to decreasing overall costs as DiMasi found that clinical approval success rates have decreased over the past decade while costs of clinical trials have increased [11]. Specifically, clinical R&D costs rose from US$608 million in the 1990’s to US$1.46 billion in the following decade. Both large and small pharmaceutical firms would benefit from decreased regulatory burden if it led to lower clinical trial costs with higher success rates. Smaller enterprises may be more prone to share early stage development or clinical data to cut overall costs. It is unclear however if larger corporations would be willing to do this as well.

Larger corporations will likely focus more on the overall long term profit of the compound, as their resources will allow them to spend a significant amount on early development and clinical trials if profitability is relatively assured. Smaller firms do not have this luxury, and require more help at earlier stages, but do not require as much profitability in the longer term. Large corporations need profits of US$800 million per compound to make a project viable, while smaller firms may need as little as US$100–200 million [13]. The IDFF addresses this discrepancy in the funding needs of large and small firms by allowing loans of up to US$75 million to one or two large pharmaceutical firms. The O’Neill group advocates for harnessing market forces to attract participation of large firms by creating large prizes, effectively assuring a market for the antibiotic, through the use of a global purchaser. This mechanism would favor participation by larger firms as opposed to smaller ones, as large firms have access to capital that would allow them to fund costly clinical trials as long as a profitable market can be guaranteed upon approval.

### Facilitate synergy

The current information available regarding the IDFF proposal does not address the concept of synergy among many stakeholders, other than to require a proportion of the funding to come from outside donors. There is no mechanism to increase collaboration among developers or affect change in regulatory structure. In contrast, the O’Neill report advocates for harmonization of the regulatory approval process across countries to minimize duplication, time requirements and cost of regulatory filings. A global approval process could help improve access to antibiotics and decrease the overall cost to the developer. Similarly, involvement of national agencies in facilitating clinical trials for antibiotics could reduce costs and speed up the approval process, as discussed in the O’Neill report.

### Public health objectives

Any effort to increase the antibiotic pipeline will align with global public health objectives, however regional priorities may differ. The IDFF requires that recipients of such grants be established or operating in member states, and that projects “must have proven public health impact and potentially market impact”. The program is aimed at vaccines, drugs and medical devices in the field of infectious diseases. It does not explicitly state that the public health needs must address those of the member states, however it is unclear if funders would be willing to pay for projects addressing regional objectives outside of the member states.

The O’Neill proposal is more broad in its scope. It seeks to establish a global market for pharmaceuticals meeting pre-determined needs. There is no restriction on the location of participating entities or their intended targets. Their contention is that a single public purchaser will be more suited to effectively address global needs and respond to changing priorities. This system would also help to limit over-production and over-prescribing of antibiotics if the supply was controlled by a single global buyer. However, this also has the potential to restrict access to the pharmaceutical, whether appropriate or not.

### Implementation feasibility

Implementation of any strategy will ultimately depend on obtaining sufficient funding. A risk sharing loan scheme such as that proposed by the IDFF would be relatively straight forward to implement and only require a committee to evaluate potential projects, as well as a sufficiently large amount of capital. Choosing successful projects will be more challenging, as there is always some asymmetry of information between applicants and funders. Still, from a practical standpoint, such a mechanism would be simple to implement.

The structure of the O’Neill proposal is a bit more complicated. The “blue sky” innovation fund would need a structure similar to that proposed by the IDFF, a committee of qualified personnel to decide on which projects to invest in. Creation of a global buyer would require a similar committee as well as a significant source of funding. Once the antibiotic has been purchased, it would then need to be appropriately distributed/allocated to member states. Mechanisms would need to be created to identify appropriate use and prevent waste/over-prescribing, as market forces would likely not be effective. Similarly, harmonization of global regulatory requirements would require a significant political effort to coordinate disparate agencies across more than a hundred countries. Sharing of research ideas and technical expertise could be accomplished quite easily through an electronic repository, but intellectual property concerns and protection could make this difficult to practically implement. Therefore, the complexity and scope that makes the O’Neill report comprehensive also makes it significantly more challenging to implement.

## Discussion

While we commend the IDFF proposal for its attempt to enhance early financing, it lacks a comprehensive strategy for stimulation of antibiotic development and relies in large part on the co-funding of projects by third parties to supplement IDFF funds. The proposal appears to require this funding and loan repayments to generate the 5-10x multiplier effect. The validity of this approach is unclear, particularly if it is dependent on repayments from projects that are generally considered to be non-viable from a commercial standpoint. Essentially, this financing facility provides cheap money to pharmaceutical corporations in an effort to lower their cost of capital and promote investment in research and development. Unfortunately, the mechanism has no clawback provision to regain additional funds or discount the costs of the drugs if they are profitable. At best, the facility recoups the cost of the loan; at worst, the loan becomes a grant to the failed enterprise. This structure’s effects on the sustainability of the financing facility and on its ability to promote true innovation are unclear. The IDFF is demand driven, in that it funds proposals put forth by industry, with no clear agenda of its own, other than a generalized mandate. Without a clear framework to identify and set priorities, there is a risk that the facility will fund priorities of corporations instead of public health objectives. The European Innovative Medicines Initiative (IMI) provides a cautionary tale of how corporate incentives may diverge from public goals. The IMI is a jointly funded initiative by the European Union and the European pharmaceutical industry. The most recent program, funded in 2014, has a budget of €3.276 billion [14], with almost half of this coming from in-kind contributions from the pharmaceutical industry. While the focus of the program is to provide early stage development funding for a variety of medicines, including antibiotics, it has come under criticism recently for lack of transparency and diversion of resources to projects felt to be lucrative by the industry, but not aligned with major European public health objectives [15, 16].

The IMI is not without its merits, and a complete discussion of this initiative is beyond the scope of this article. It does serve a useful mechanism as early stage funding for development of compounds, much the same way that the IDFF proposal could fund products in clinical testing. While these initiatives (IMI and IDFF) have the potential to be complementary, no framework exists to facilitate this, leaving an overall disjointed response to funding innovation.

Regardless of the mechanism employed, giving away cheap money to corporations is the low hanging fruit in spurring development: it is simple and relatively easy to implement and monitor. These payments are crucial to the overall success of any plan, as incentives will be most effective when companies are partially shielded from losses with lump sum payments, but still have potential for market gains if the product proves to be profitable. Therefore, the crucial question is the timing of the lump sum payment. The simplest method would involve a large payment at the time of market approval, providing a reward for successful development, or a large purchase to help control global market share. While the prospect of making large payments at the time of market approval is attractive because it allows the buyer to know exactly what they are purchasing, and thus fulfills the goal of good stewardship of public funds, it is short sighted and flawed, for the reasons previously discussed.

Regardless of the timing of a potential payment, investments at any stage should come with accountability. Conditions should be given to any developer receiving funding from the O’Neill report’s push mechanism. This will likely be less palatable to large pharmaceutical corporations, but better tolerated by small to medium sized enterprises who may otherwise lack the capital needed to bring a novel drug to market. Zero risk loans are highly desired by the pharmaceutical industry, and given their political clout, we fear that this may sway policy makers to structure push mechanisms as forgivable loans (as is proposed by the IDFF). This would not be a judicious use of public funds.

We have previously proposed an alternative model, the options market for antibiotics [17, 18], which is a hybrid of early incentive payments combined with payments at the time of approval, similar to the hybrid model described by the O’Neill report. This model calls for a single payment or series of payments at any stage of development, in exchange for the promise of discounts to purchasers if and when the drug makes it to market. It is modeled on a financial call option, which allows investors to speculate on the chances of a stock rising or falling. The early payout helps to defray development costs, stimulating research and development by small and large companies alike. The discount at the time of market approval helps the payer efficiently purchase the new compound. Even more importantly, the early payout will allow funders to get more “bang for the buck”, as early stage investments need not be as large to have the same impact as late stage funding. Other hybrid models have been developed in recent years with similar components. The Project BioShield Act in the US provided a guaranteed government market for medical countermeasures to treat biological threats, as well as federal funding to stimulate research [19]. The World Health Organization Global Consortium model utilizes milestone prizes, research grants, open source data sharing and advance purchase agreements to stimulate neglected antimicrobial development [20]. These two examples demonstrate that a unified solution must have provisions to stimulate and reward early development, as well as provide a viable market after approval has been granted.

Of course, the key to any of these proposals is a large financing facility that has the resources and freedom to invest in potentially uncertain projects. The O’Neill Review advocates an innovation fund to support “blue sky” scientific research. We agree that this is crucial, yet it need not be separate from the goal of creating a predictable market. Push and pull mechanisms can be combined, but a credible payer must be able to commit early, as well as demonstrate a capacity to reward innovators at late stages. This requires money, and expertise in judging promising projects. A fund such as that proposed by the IDFF may prove well suited to just such a task.

Overall, the aims of the IDFF proposal are noble, but incomplete on their own. The O’Neill report goes further with recommendations regarding more comprehensive policy reform to address market imperfections. Neither goes far enough in demanding some type of tradeoff from manufacturers should the drug be successful (beyond requiring loan repayment). Investment is risky, and it is not unreasonable to expect investors (even public ones) to be compensated for this risk in the form of lower prices or clawback provisions. This could ensure the continued feasibility of such a fund, particularly if the clawback payments went towards further funding. However, such an idea will likely be unpopular and politically difficult to pass. Developers may argue that such a provision will hurt innovation, however it is an axiom of modern finance that risk can be calculated, and appropriately compensated for.

Given the political sensitivity of such an issue, this demands further attention from a global body, such as the G20. Any single national government could be easily coerced by the pharmaceutical lobby. However, when dealing with a problem of international market failures, an international solution is necessary. Therefore we strongly urge the G20 to consider the above proposals as a framework to begin drafting a unified solution to the impending threat of antimicrobial resistance at their next summit. A model for this may be found in the efforts of the European Union’s Joint Programming Initiative on Antimicrobial Resistance (JPIAMR), which works to coordinate the efforts of EU funded research projects on antimicrobial resistance [21]. A G20 version of this body may prove to be perfectly suited to coordinate global programs on the issue.

## Conclusions

Ultimately, the success or failure of any effort to stem the tide of antimicrobial resistance and develop new antibiotics will hinge on the ability to coordinate a global effort. Large global funders may have the market share and regulatory powers to both incentivize development and control reckless distribution. We believe a hybrid model of early payments, a strong commitment by credible purchasers, and the ability to sell on the open market at the time of drug approval are crucial, but above all, a unified global response is essential. The last three months have shown two commendable efforts arise in Europe, but even these are currently disjointed in their implementation. Well-intentioned, elegant strategies executed in a disorganized fashion will do little to confront the impending crisis. The global health community will be better served by coordinating efforts between the IDFF as a financing mechanism and the comprehensive guidance of the O’Neill report.

### Ethics approval and consent to participate

Not applicable.

### Consent for publication

Not applicable.

## Abbreviations

IDFF: International Disease Financing Facility
EIB: European Investment Bank
EC: European Commission
SME: small to medium sized enterprises
NPV: net present value
NME: new molecular entities
IMI: innovative medicines initiative
JPIAMR: Joint Programming Initiative on Antimicrobial Resistance

## References

[1] O’Neill J. Antimicrobial resistance: tackling a crisis for the health and wealth of nations. Rev Antimicrob Resist. 2014. http://amr-review.org/Publications.

[2] Mossialos E, Morel CM, Edwards S, Berenson J, Gemmill-Toyama M, Brogan D (2010). Policies and Incentives for Promoting Innovation in Antibiotic Research.

[3] Matthiessen L (2015). Infectious Disease Financing Facility (IDFF). Horizon 2020 SC1 Advisory Group for the “Health, demographic change and well-being”.

[4] O’Neill J (2015). Securing New Drugs for Future Generations: The Pipeline of Antibiotics.

[5] Renwick M, Brogan D, Mossialos E. A Critical Assessment of Incentive Strategies for Development of Novel Antibiotics. J Antibiot. 2015; doi: 10.1038/ja.2015.98 10.1038/ja.2015.98 PMC4775540 26464014

[6] Morel CM, Mossialos E (2010). Stoking the antibiotic pipeline. BMJ.

[7] Sharma P, Towse A (2010). New drugs to tackle antimicrobial resistance: analysis of EU policy options.

[8] Munos B (2009). Lessons from 60 years of pharmaceutical innovation. Nat Rev Drug Discov.

[9] Cecchini M, Langer J, Slawomirski L (2015). Antimicrobial Resistance in G7 Countries and Beyond: Economic Issues, Policies and Options for Action.

[10] Harper M. The Truly Staggering Cost of Inventing New Drugs. Forbes, 2012. Available from: www.forbes.com/sites/matthewherper/2012/02/10/the-truly-staggering-cost-of-inventing-new-drugs/#2715e4857a0b70d756604477. Accessed on October 7, 2015.

[11] DiMasi J (2014). Innovation in the Pharmaceutical Industry: New Estimates of R&D Costs.

[12] Avorn J (2015). The $2.6 Billion Pill—Methodologic and Policy Considerations. N Engl J Med.

[13] Monnet DL (2005). Antibiotic development and the changing role of the pharmaceutical industry. Int J Risk Safety Med.

[14] Innovative Medicines Initiative (2015). The IMI funding model Innovative Medicines Initiative.

[15] Elmer C, Grossenbacher T, Gruhnwald S, Schafer M (2015). The People Pay, Corporations Cash In: Problems Plague EU Medical Research Initiative. Der Spiegel.

[16] Garattini S, Bertele V, Bertolini G (2013). A failed attempt at collaboration. BMJ.

[17] Brogan D, Mossialos E (2006). Applying the concepts of financial options to stimulate vaccine development. Nat Rev Drug Discov.

[18] Brogan DM, Mossialos E (2013). Incentives for new antibiotics: the Options Market for Antibiotics (OMA) model. Glob Health.

[19] Russell PK (2007). Project BioShield: what it is, why it is needed, and its accomplishments so far. Clin Infect Dis.

[20] World Health Organization (2014). Publicly Financed Global Consortium for R&D to Fight Antibiotic Resistance.

[21] Joint Programming Initiative on Antimicrobial Resistance (2013). Strategic Research Agenda.

